# Interannual fluctuations in connectivity among crab populations (*Liocarcinus depurator*) along the Atlantic-Mediterranean transition

**DOI:** 10.1038/s41598-022-13941-4

**Published:** 2022-06-13

**Authors:** Víctor Ojeda, Bruna Serra, Clàudia Lagares, Eva Rojo-Francàs, Maria Sellés, Elena Marco-Herrero, Encarnación García, Marc Farré, Concepció Arenas, Pere Abelló, Francesc Mestres

**Affiliations:** 1 Dept. Genètica, Microbiologia i Estadística. Secció de Genètica Biomèdica, Evolució i Desenvolupament, Universitat de Barcelona, Av. Diagonal, 643, 08028 Barcelona, Spain; 2 IRBio (Institut de Recerca Per la Biodiversitat), Universitat de Barcelona, Barcelona, Spain; 3 Instituto Español de Oceanografía - C.O. Cádiz (IEO-CSIC), Cádiz, Spain; 4 Instituto Español de Oceanografía - C.O. Murcia (IEO-CSIC), San Pedro del Pinatar, Spain; 5 Instituto Español de Oceanografía - C.O. Balears (IEO-CSIC), Palma de Mallorca, Spain; 6 Dept. Genètica, Microbiologia i Estadística, Secció d’Estadística, Universitat de Barcelona, Barcelona, Spain; 7 Institut de Ciències del Mar (ICM-CSIC), Barcelona, Spain

**Keywords:** Evolution, Genetics, Zoology

## Abstract

An interesting evolutionary question that still remains open is the connectivity between marine populations. Marine currents can favour the dispersal of larvae or adults, but they can also produce eddies and gyres generating oceanographic fronts, thus limiting gene flow. To address this subject, we selected the Atlantic-Mediterranean transition, where several fronts are located: Gibraltar Strait (GS), Almeria-Oran Front (AOF) and Ibiza Channel (IC). Seven populations of the marine crab *Liocarcinus depurator* (Cadiz, West and East Alboran, Alacant, Valencia, Ebro Delta and North Catalonia) located along this transition were analysed in six consecutive years (2014–2019) using a fragment of the *COI* (*Cytochrome Oxidase subunit I*) gene. All sequences (966) belonged to two well defined haplogroups: ATL (most abundant in Atlantic waters) and MED (predominant in Mediterranean waters). Following a geographic variation, the frequency of ATL decreased significantly from Cadiz to North Catalonia. However, this variation presented steps due to the effect of oceanographic restrictions/fronts. Significant effects were recorded for GS (2015, 2017, 2018 and 2019), AOF (all years except 2018) and IC (2016). The intensity and precise location of these fronts changed over time. Multivariate analyses distinguished three main population groups: Cadiz, Alboran Sea and the remaining Mediterranean populations. These findings could be relevant to properly define Marine Protected Areas and for conservation and fisheries policies.

## Introduction

A superficial/preliminary appraisal would tend to suppose that many marine species (in adult or larval forms) could freely move, even far away, in the water mass. These movements would generate a high gene flow, the consequence of which would be a low population differentiation. Under this assumption, distance would be the only factor generating isolation/genetic differentiation between marine populations. However, this is not the actual situation, since available, relatively recent information on the occurrence of oceanographic barriers has been accumulating. These fronts are the product of physical movements of the water masses and can significantly restrict the movement and spread of marine species^1–5^. Additionally, marine currents and counter currents, eddies and other displacements in the three-dimensional marine environment can largely affect not only larval dispersion, but also the distribution of juveniles or adults, thus transporting them at great distances from where parental spawning took place. These processes would thus generate appreciable gene flows^6,7^. Accordingly, connectivity among populations would highly depend on a balance between the gene flow generated by dispersing currents, and marine fronts preventing it. Furthermore, these oceanographic processes are temporally dynamic and generate high levels of temporal variability in connectivity patterns. Also, behavioural factors, especially in the pelagic larval phase, may be relevant in connectivity.

Although oceanographic fronts have been described in all world oceans and seas^3^, the Mediterranean Sea is especially adequate to study these processes due to its specific physical and biological processes at play in this area. The present Mediterranean oceanographic conditions are the result of both geological events (historic factors) and contemporary processes, which together shape the biodiversity patterns that are responsible of the present marine species distributions and of the genetic variability observed in their populations^8^. All these processes have contributed to produce and shape the rich biodiversity currently present in the Mediterranean Sea, which is considered a biodiversity hotspot^9–12^.

This sea is a semi-enclosed basin connected with the Atlantic Ocean by the Gibraltar Strait and divided in two main sub-basins (Western and Eastern) by the Sicily Strait^13^. It is also connected with the Red Sea via the Suez Canal. The Mediterranean Sea receives a large amount of solar radiation, low input of freshwater, and the winter northerly winds (for instance, Mistral, Tramontana and Bora) are characteristically very dry and cold^14,15^. All these factors combine to produce a substantial evaporation of surface water in both winter and summer, which thus implies a remarkable increase in salinity, in addition to a remarkable decrease in temperature in winter. These processes very often imply that the large increase in density (due to the combined effect of increasing salinity and decreasing temperature) contributes to the winter advection of these waters towards the bottom in some areas^16^. It is worth remarking that in the latter case, these processes have also the important biological implication of transporting oxygen to the deep sea. As a consequence of both winter evaporation (due to the above-mentioned processes), and summer evaporation due to solar irradiation, coupled with a remarkable decrease of river inputs due to industrialization and damming, the Mediterranean basin suffers from an important water deficit, which equilibrium is mainly supported by the epipelagic influx of Atlantic water (due to its lower density) through the Strait of Gibraltar. It then circulates along the northern shores of the Alboran Sea until Almeria/Cape Gata, where it heads then southeast towards the North African coasts^17^. This movement generates two anticyclonic gyres^18^, the western (WAG) being usually stronger and more stable than the eastern (EAG)^19–21^. The current follows along the African coast until Sicily (Algerian current), where one of its branches is deviated to the North of Italy, South of France and North of the Spanish coasts (the Liguro–Provencal–Catalan or Northern current) until the Ibiza Channel^22^. There it usually continues southwest, but, due to particular winter conditions in some years, it may recirculate northwards along the western coasts of the Balearic Islands, thus reinforcing the Balearic current and creating a temporal cyclonic gyre in the basin^23^. The other branch of the Atlantic jet flows to the Eastern Mediterranean^14^. Thus, the dynamics of all these water masses and their circulation generate several oceanic barriers in the Western basin, such as the Gibraltar Strait (GS), the Almeria-Oran Front (AOF), the Ibiza Channel (IC) and the Balearic Front (BF)^5^.

**Figure 1:**
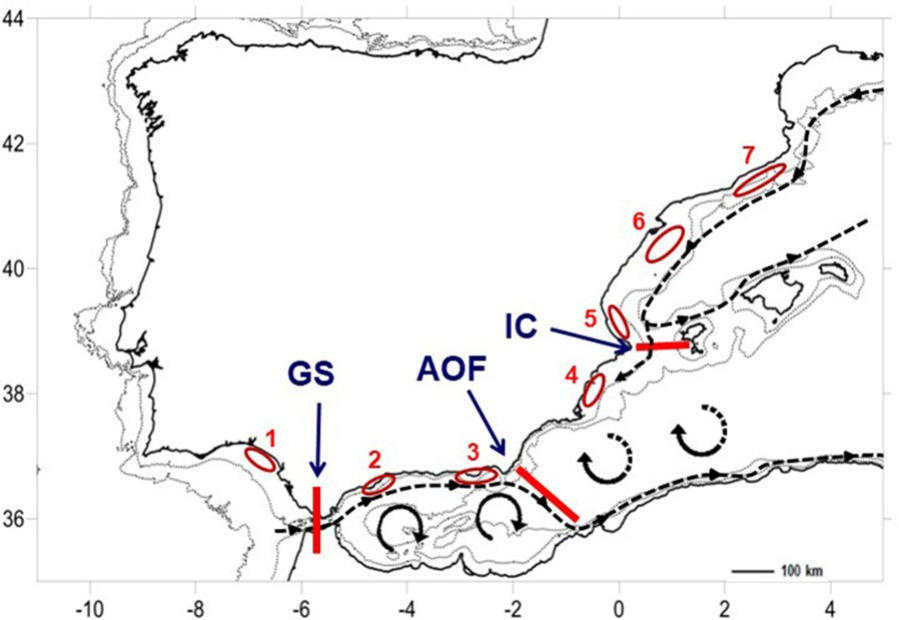
Sampling populations of *Liocarcinus depurator* in the Atlantic-Mediterranean transition. Most populations were sampled in the period 2014–2019 (see text for details). Sampling areas appear in red colour. Location numbers and acronyms are: 1. Cadiz (CADI), 2. West Alboran Sea (WALB), 3. East Alboran Sea (EALB), 4. Alacant (ALAC), 5. Valencia (VALE), 6. Ebro Delta (DELT) and 7. North Catalonia (NCAT). Dashed lines identify the main currents and gyres in the studied area. Oceanographic fronts appear as solid red lines: Gibraltar Strait (GS), Almeria-Oran Front (AOF) and Ibiza Channel (IC). This figure was prepared with the help of Surfer (Golden software Inc.) (https://www.goldensoftware.com/products/surfer).

The main aim of this research was to study the connectivity between populations of benthic animals as well as to analyse how gene flow is affected by the oceanographic processes. Furthermore, and principally, we wanted to study the possible temporal variation in the connectivity patterns due to fluctuations of the oceanographic discontinuities. To this purpose, we focused on the Atlanto-Mediterranean transition along the Iberian Peninsula coasts to study the effect of GS, AOF and IC in the gene flow among populations of the marine crab *Liocarcinus depurator* (Linnaeus, 1758) (Fig. 1). As adult, this portunid crab lives on muddy sea bottoms of the continental shelf and upper continental slope, from shallow continental shelf waters to over 300 m depth, being one of the most common epibenthic crustaceans of the studied region^24–26^. The species presents sexual dimorphism and its reproductive period in Mediterranean populations takes mainly place during autumn–winter, although ovigerous females can be recorded throughout the year^27,28^. The fecundity of *L. depurator* is relatively high, from 10,000 to over 200,000 eggs *per* female^28^ and the eggs are carried on the female abdomen during approximately four weeks, depending on the temperature^29^. Larval development consists of two phases: five zoea stages and one megalopa stage^30,31^. Early zoeas (the dispersal phase) are epipelagic and freely disperse until megalopas (recruitment phase) settle on the continental shelf bottom^32^. Planktonic larval duration, although highly dependent on temperature, is estimated to be of at least five weeks^33,34^. Juvenile crabs grow rapidly, and sexual maturity is reached during their first year of life^27,28^. For these reasons, the adult individuals observed in a particular population and year are mainly dependent on the recruitment from the previous year.

The selection of *L. depurator* as an appropriate species to perform this study is also a consequence of the specific characteristics of a useful genetic marker, a fragment of the mitochondrial *COI* (*Cytochrome Oxidase subunit I*) gene. Previous observations detected that *L. depurator* haplotypes could be clearly classified in two well-differentiated groups: one predominant in Atlantic waters, whereas the other is mainly detected in Mediterranean waters^8,35^. Accordingly, this species was chosen as an adequate organism to test whether gene flow between populations is affected by oceanographic fronts, since it is also commonly distributed along the whole region of interest (the Atlanto-Mediterranean transition). Furthermore, it presents a very valuable genetic marker to study the connectivity processes and interannual variability between their populations.

The hypothesis of our research was based on whether oceanographic fronts condition the connectivity among *L. depurator* populations. Using the *COI* gene fragment, the two main aims of our research were to estimate the genetic structure and the connectivity of seven populations of *L. depurator* distributed along the Atlanto-Mediterranean transition (Cadiz, West Alboran, East Alboran, Alacant, Valencia, Ebro Delta, and North Catalonia). Accordingly, we intended to assess the effect of the marine barriers located in this area (Gibraltar Strait, Almeria-Oran Front and Ibiza Channel) on the gene flow between populations. For this objective, the selected populations were located on each side of these barriers. Thus, Gibraltar Strait is placed between Cadiz and West Alboran, the Almeria-Oran Front is flanked by East Alboran and Alacant, and the Ibiza Channel is located between Alacant and Valencia. Acting as controls, two populations not affected by any barrier, Ebro Delta and North Catalonia, were also analysed. Finally, since these oceanographic fronts are temporally semi-permanent and their precise placement is relatively dynamic over time^23,36,37^, it was assumed that their intensity and precise placement may be useful to elucidate to what extent their variations could influence the connectivity levels between populations estimated from crab populations surveys along six consecutive years (2014–2019).

## Results

The parameters estimating the molecular diversity of the 966 *L. depurator* sequences obtained according to population and year are presented in Table S1. The list of 118 different detected haplotypes and their distribution according to population and year is presented in Table S2. The most frequent haplotypes were Ldep_02 and Ldep_03, whilst 86 haplotypes were found only once. In general, WALB showed the highest molecular diversity, although the highest values of haplotype and nucleotide diversity corresponded to CADI 2016 (0.872 ± 0.091 and 0.535 ± 0.673, respectively). The smallest estimates corresponded to VALE (0.145 ± 0.090 and 0.056 ± 0.040, respectively).

Concerning the networks computed with the haplotypes, either studying all sequences together (Fig. S1) or separately by year (data not shown), similar results were always obtained. Thus, a couple of star-shape groups, each centred on a highly frequent haplotype (Ldep_03 and Ldep_02), was observed. In general, the haplotypes belonging to Ldep_03 and related haplotypes were located in areas with a strong Atlantic influence such as the Gulf of Cadiz and the coasts of the Alboran Sea (ATL), whereas the individuals of the group centred on Ldep_02 and related haplotypes, were most commonly found east of the Alboran Sea, in fully Mediterranean locations (MED). Moreover, using the same haplotypes, the phylogenetic trees obtained for each year (data not shown) or using all the different haplotypes detected for this gene fragment together (Fig. S2) always showed the same topology: a clear partition separating the haplotypes of ATL group (Ldep_03 and related) from those of the MED group (Ldep_02 and related). These results agree with those from previous research in the study area^8,35^, thus confirming the occurrence of two stable haplogroups. In the present research, 64 out of 118 different described haplotypes belonged to the ATL haplogroup, whilst 54 to the MED haplogroup. All four sequences reported from IALB (Alboran Island) belonged to the ATL group.

The relative abundance of the ATL haplogroup in each studied population and year is presented in Table 1, which shows that the genetic composition of the populations fluctuated over time. For each population, the differences for each pair of consecutive years, and globally for the whole period were analysed by computing the corresponding Gamma_ST_ and Snn values along with their respective P values (Table 2). For the Atlantic CADI population, three particular comparisons (2014–2017, 2015–2016 and 2015–2017) were significant and also the overall comparison considering all years together. This significance was however lost after statistical adjustment for multiple testing. Remarkably, the EALB comparisons between 2016–2017 and 2017–2018 were significant, and the former significance remained after statistical adjust. Finally, two comparisons were significant in DELT (2015–2018 and 2016–2018), although these significances were lost after correction.

**Table 1:** Frequency of the ATL haplogroup in the studied populations over time.

Year	Populations						
	CADI	WALB	EALB	ALAC	VALE	DELT	NCAT
2014	0.852 (0.663, 0.958)	0.828 (0.642, 0.942)	‒	0.100 (0.021, 0.265)	0.065 (0.008, 0.214)	0.138 (0.039, 0.317)	‒
2015	0.963 (0.810, 0.999)	0.600 (0.406, 0.773)	‒	0.100 (0.012, 0.317)	0.133 (0.038, 0.307)	0.200 (0.077, 0.386)	‒
2016	0.692 (0.386, 0.909)	0.667 (0.447, 0.844)	0.131 (0.028, 0.336)	0.160 (0.044, 0.349)	0.024 (0.001, 0.129)	0.120 (0.025, 0.312)	0.0
2017	0.737 (0.488, 0.909)	0.500 (0.313, 0.687)	0.800 (0.519, 0.957)	0.143 (0.040, 0.327)	0.077 (0.009, 0.251)	0.120 (0.025, 0.312)	0.0
2018	0.875 (0.710, 0.965)	0.500 (0.313, 0.687)	0.355 (0.192, 0.546)	0.129 (0.036, 0.298)	0.100 (0.021, 0.265)	0.034 (0.001, 0.178)	0.0
2019	0.929 (0.765, 0.991)	0.517 (0.325, 0.706)	0.429 (0.245, 0.628)	0.074 (0.009, 0.243)	0.037 (0.001, 0.190)	0.208 (0.071, 0.422)	0.074 (0.009, 0.243)

For each value, interval of confidence is presented in the row below between brackets. The population acronyms are: CADI (Cadiz), WALB (West Alboran Sea), EALB (East Alboran Sea), ALAC (Alacant), VALE (Valencia), DELT (Ebro Delta) and NCAT (North Catalonia). No samples were collected in 2014 and 2015 in EALB and NCAT.

**Table 2:** Gamma_ST_ and *Snn* values with the corresponding P values computed in each population for each pair of consecutive years.

Population	Years compared	Gamma_ST_	*Snn*	P	Adjusted *P*	Gamma_ST_	Snn	P	Adjusted P
CADI	2014–2015 2014–2016 2014–2017 2014–2018 2014–2019 2015–2016 2015–2017 2015–2018 2015–2019 2016–2017 2016–2018 2016–2019 2017–2018 2017–2019 2018–2019	0.023 0.027 0.043 0.013 0.025 0.066 0.089 0.015 0.028 0.024 0.027 0.034 0.043 0.037 0.018	0.514 0.559 0.608 0.501 0.573 0.623 0.610 0.500 0.523 0.574 0.578 0.596 0.592 0.519 0.520	0.293 0.419 **0.030** 0.432 0.052 **0.042** **0.007** 0.456 0.155 0.096 0.523 0.204 0.091 0.332 0.256	0.440 0.489 0.195 0.489 0.195 0.195 0.105 0.489 0.332 0.240 0.523 0.382 0.240 0.453 0.427	0.052	0.207	**0.019**	0.114
WALB	2014–2015 2014–2016 2014–2017 2014–2018 2014–2019 2015–2016 2015–2017 2015–2018 2015–2019 2016–2017 2016–2018 2016–2019 2017–2018 2017–2019 2018–2019	0.029 0.022 0.063 0.065 0.061 0.010 0.022 0.023 0.018 0.025 0.029 0.020 0.005 0.005 0.005	0.508 0.491 0.540 0.522 0.514 0.471 0.550 0.520 0.491 0.520 0.523 0.435 0.482 0.462 0.457	0.326 0.515 0.129 0.162 0.234 0.709 0.082 0.235 0.439 0.300 0.183 0.926 0.595 0.798 0.853	0.611 0.770 0.587 0.587 0.587 0.886 0.587 0.587 0.732 0.611 0.587 0.926 0.811 0.914 0.914	0.044	0.173	0.202	0.306
EALB	2016–2017 2016–2018 2016–2019 2017–2018 2017–2019 2018–2019	0.332 0.052 0.082 0.120 0.089 0.009	0.710 0.552 0.522 0.608 0.568 0.484	**0.000** 0.072 0.186 **0.027** 0.115 0.635	**0.000** 0.144 0.223 0.081 0.173 0.635	0.132	0.268	0.271	0.325
ALAC	2014–2015 2014–2016 2014–2017 2014–2018 2014–2019 2015–2016 2015–2017 2015–2018 2015–2019 2016–2017 2016–2018 2016–2019 2017–2018 2017–2019 2018–2019	0.010 0.009 0.020 0.011 0.011 0.012 0.020 0.009 0.012 0.017 0.012 0.014 0.011 0.026 0.011	0.472 0.517 0.538 0.467 0.488 0.508 0.503 0.460 0.458 0.543 0.516 0.500 0.490 0.499 0.440	0.839 0.279 0.069 0.756 0.506 0.336 0.501 0.928 0.864 0.089 0.237 0.397 0.646 0.326 0.958	0.958 0.840 0.668 0.958 0.958 0.840 0.843 0.958 0.958 0.668 0.840 0.843 0.958 0.840 0.958	0.023	0.175	0.204	0.306
VALE	2014–2015 2014–2016 2014–2017 2014–2018 2014–2019 2015–2016 2015–2017 2015–2018 2015–2019 2016–2017 2016–2018 2016–2019 2017–2018 2017–2019 2018–2019	0.014 0.012 0.009 0.010 0.011 0.030 0.013 0.009 0.029 0.015 0.019 0.007 0.010 0.011 0.017	0.491 0.509 0.497 0.482 0.499 0.526 0.483 0.494 0.520 0.518 0.493 0.494 0.502 0.473 0.487	0.499 0.345 0.570 0.582 0.607 0.225 0.585 0.462 0.201 0.564 0.659 0.963 0.423 0.590 0.587	0.700 0.700 0.700 0.700 0.700 0.700 0.700 0.700 0.700 0.700 0.706 0.963 0.700 0.700 0.700	0.025	0.164	0.569	0.569
DELT	2014–2015 2014–2016 2014–2017 2014–2018 2014–2019 2015–2016 2015–2017 2015–2018 2015–2019 2016–2017 2016–2018 2016–2019 2017–2018 2017–2019 2018–2019	0.009 0.023 0.013 0.024 0.017 0.019 0.013 0.042 0.013 0.015 0.034 0.035 0.020 0.024 0.050	0.492 0.518 0.499 0.499 0.509 0.505 0.495 0.536 0.535 0.483 0.542 0.554 0.468 0.506 0.542	0.298 0.168 0.318 0.367 0.305 0.251 0.410 **0.045** 0.092 0.514 **0.049** 0.066 0.704 0.332 0.104	0.453 0.420 0.453 0.459 0.453 0.453 0.473 0.312 0.312 0.551 0.312 0.312 0.704 0.453 0.312	0.038	0.175	0.166	0.306

The population acronyms are the same as those presented in Table 1. Significant P values appear in bold.

**Figure 2:**
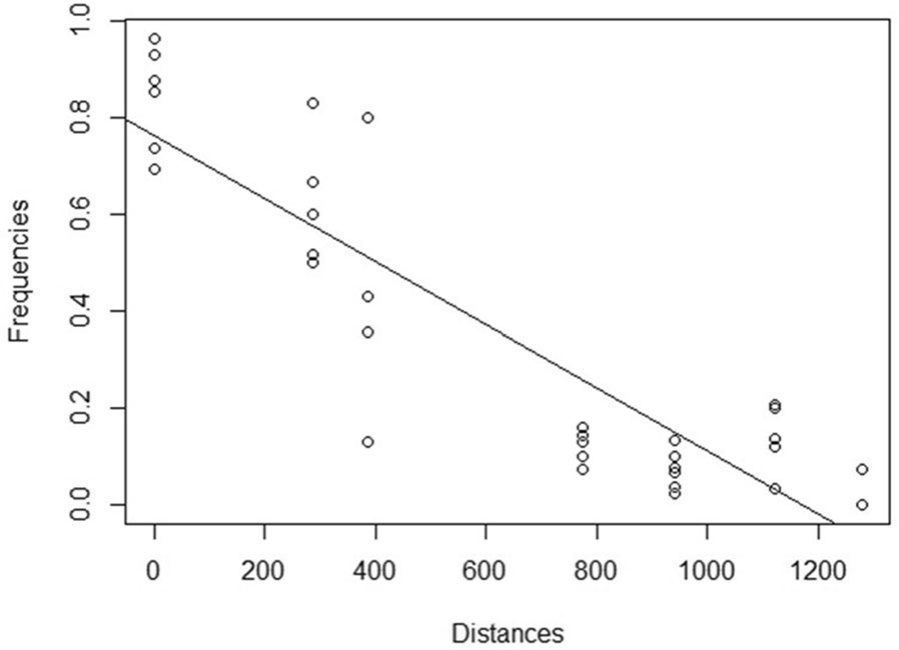
Linear regression model between the frequency of ATL haplogroup and geographic distance considering all years of the research. The model showed a significant decrease in ATL frequency when the geographic distance increases.

**Figure 3:**
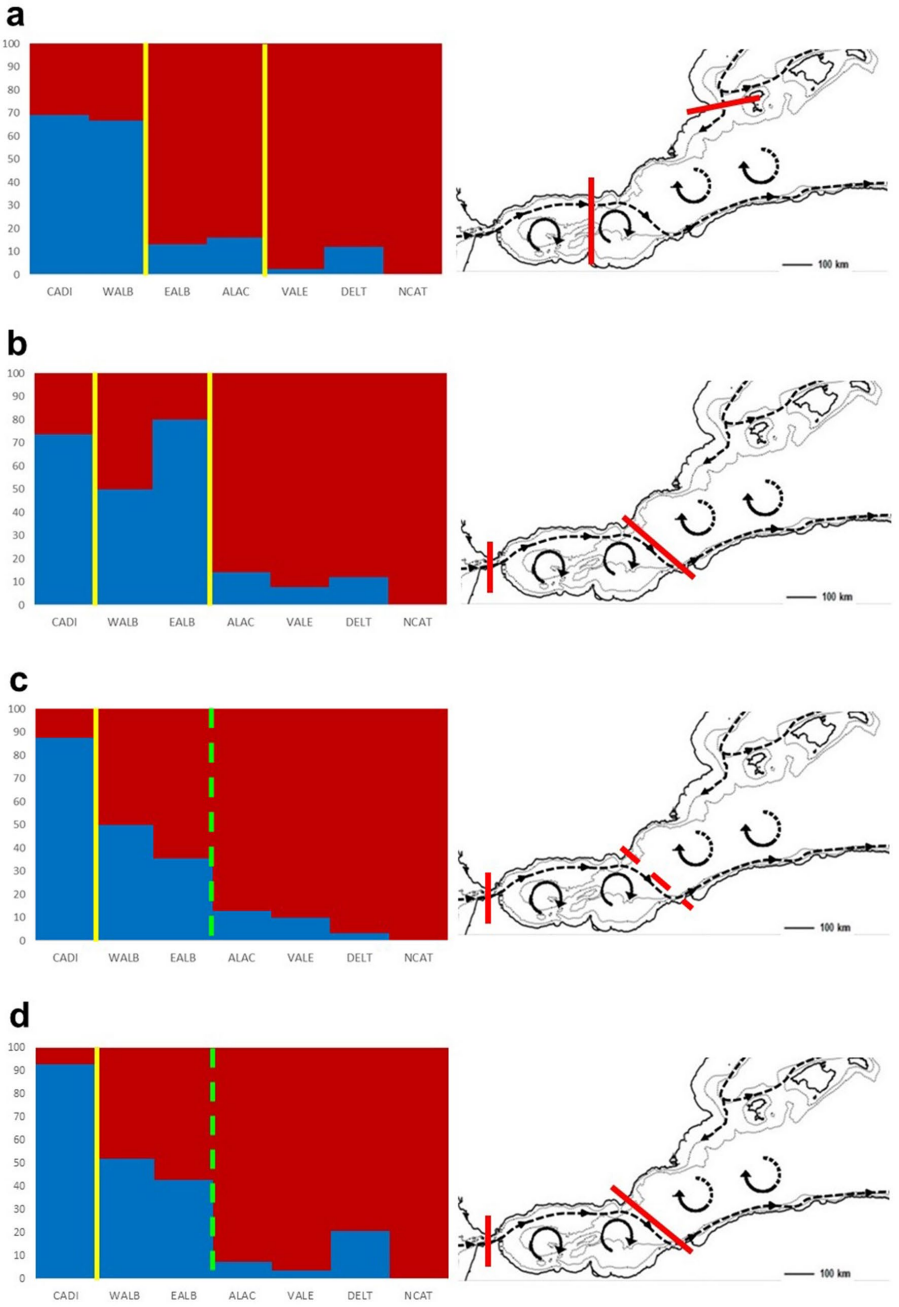
Percentages of ATL (blue) and MED (red) haplogroups in the populations of the Atlantic-Mediterranean transition, differences significant between populations and detection of the oceanographic front’s effects. For each year, the differences in haplogroup frequencies between neighbouring populations are presented (continuous yellow lines and dashed green lines stand for significant and a trend of differentiation, respectively). Each histogram is accompanied by a map showing the effect of the corresponding oceanographic front, that could be intense (continuous red lines, dashed red lines stand for significant and trend effect of the oceanographic front, respectively). (**a**) 2016, (**b**) 2017, (**c**) 2018 and (**d**) 2019. The population acronyms are the same as in Fig. 1. This figure was prepared with the help of Surfer (Golden software Inc.) (https://www.goldensoftware.com/products/surfer).

Mantel tests showed a positive relation between the genetic and geographic distances which were significant in most years (2014, r = 0.8897, P = 0.025; 2015 r = 0.8530, P = 0.050; 2016, r = 0.5225, P = 0.043; 2017, r = 0.6164, P = 0.054; 2018, r = 0.8435, P = 0.001; 2019, r = 0.7078, P = 0.011). Moreover, a variation pattern of both haplogroups was observed along the Atlantic-Mediterranean study area, the ATL haplogroup being the most frequent in CADI, while decreasing its proportion towards the populations located further inside the Mediterranean (Table 1). This pattern was quantified, and the ANCOVA model with distance and year as covariables showed that distance was the only covariable significantly related with frequencies (P values were: 1.6767e-31 for distance and 0.7726 for year). Thus, the linear regression model run for the whole dataset (Fig. 2) showed a significant decrease in frequency (F = 0.0762 − 0.0007 * D) with increasing distance (P = 2.4399e−14, ${\mathrm{R}}_{\mathrm{adj}}^{2}=0.80$). However, this variation is not continuous in space due to the occurrence of different steps that could vary over time. These steps could be related with the oceanographic discontinuities. Therefore, to study these dynamic structures (or processes), the genetic distances between neighbour populations, Gamma_ST_ and Snn values (with their respective P values) were computed, and the corresponding results presented in Table S3. It is herein worth remembering that the haplogroups distribution found for the adults of a specific year would correspond to the effects of currents and associated oceanographic fronts on the larvae of the previous year. The GS presented a significant effect in 4 out of the 6 years studied, thus constituting a highly relevant oceanographic discontinuity for *L. depurator* (Fig. 3b–d). The AOF was also a relevant front for the species since significance was high for all years when comparing WALB with ALAC, although it was lost (non-significant) in 2017 and 2018 when applying the FDR correction. Furthermore, for years when samples of EALB were available (2016–2019) the location of AOF was more precisely shown (Fig. 3). Thus, significant differences were observed between WALB and EALB in 2016, suggesting that the strongest effect was located east of the first anticyclonic gyre. In 2017, the significance between EALB and ALAC was still present after the FDR correction, thus indicating that the front was placed in its usual location. On the following year, 2018, the effect of AOF was not especially intense, since significance was only detected between WALB and ALAC, and was lost after implementation of the FDR correction. Finally, significance was observed between WALB and ALAC in 2019, suggesting a strong effect of AOF. For the IC, significance was only observed in 2016 (Fig. 3a). In the same year, a significant differentiation was also detected between VALE and DELT, although there is not any obvious discontinuity between them.

**Figure 4:**
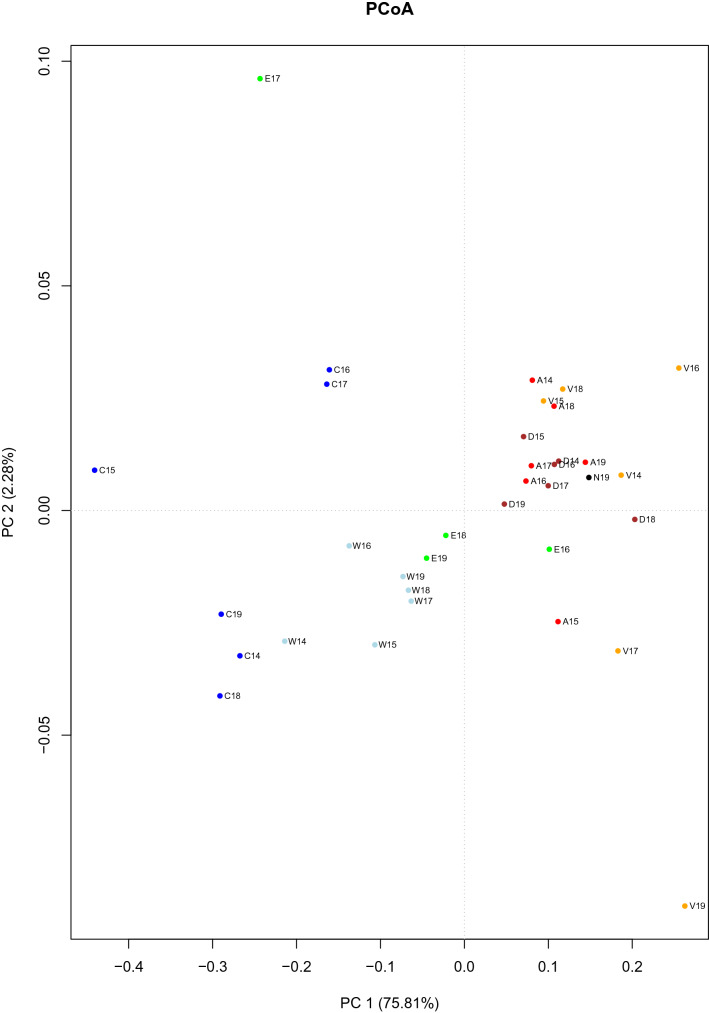
PCoA analysis using the genetic distances between all population pairs. The first and second axes explain 75.81 and 2.28% of the variability, respectively. Three main groups of population can be distinguished: Gulf of Cadiz, Alboran Sea and the remaining populations. Each population is indicated by an initial and a different colour and followed by the year (CADI: C, dark blue; WALB: W, light blue; EALB: E, light green; ALAC: A red; VALE: V, light brown; DELT: D, dark brown; NCAT: N, black).

The PCoA based on genetic distances yielded further relevant information on the distribution of the different populations (Fig. 4). The first axis explained most of the variability (75.81%), whereas the second one accounted for only 2.28%. This analysis shows that it is possible to clearly distinguish three main population groups: (1) Gulf of Cadiz, (2) Alboran Sea populations, and (3) the remaining Mediterranean populations. The first group clearly corresponds to populations with water masses of Atlantic composition where the ATL haplogroup was predominant. The Alboran Sea is an area of mixture between Atlantic and Mediterranean waters, and therefore the genetic composition for the studied *COI* fragment may undergo short and mid time considerable variations. Finally, the remaining populations, where the most abundant haplogroup is MED, inhabit areas with predominance of Mediterranean waters. However, this group did not follow a clear geographic pattern, with some DELT samples being closer than expected to the Alboran Sea populations, while VALE showed a displacement to the other extreme of the distribution.

## Discussion

In the marine benthic and epibenthic environment, species population dispersion takes place mainly during the pelagic larval stages, since larvae may travel larger distances when associated to oceanic currents^38^. Currents are one of the most important physical factors involved on these movements, and their dynamics may create frontal structures, associated or not to gyres, which may act as barriers to species dispersion. Oceanographic fronts can change quickly over time with respect to their intensity and precise geographical location, since they are dynamic structures that may be semi-permeable depending on their three-dimensional structure and short-time dynamics. All these patterns may increase variability and a certain randomness to the final regional location of the larvae when ready to metamorphose to their last larval stage (megalopa, in the case of crabs), which is the stage that performs the final settlement to the benthos. Most crab species are coastal or continental shelf dwellers^25^, but during larval development, the first zoeal stages are usually located in epipelagic waters^39,40^. Subsequent zoeal stages are usually found in deeper waters, but megalopae are again usually associated with surface or near-surface waters, as is the case of the present species^32^.

In this context, the 527 pb fragment of the *COI* mitochondrial gene of *L. depurator*, which shows a large molecular variability^8,35^, is an excellent model to try to understand the connectivity patterns among its populations along the Atlantic-Mediterranean transition, where currents and three potential oceanographic fronts condition gene flow. Likely, the larger WALB diversity could be due to the mixture of Atlantic and Mediterranean waters in the Alboran Sea region. Over the study area, the smallest diversity estimates corresponded to VALE, probably as a consequence of the engulfed waters in the collecting area, which may act as a cul-de-sac, thus generating higher levels of population isolation. Also, this population is the last of the studied areas potentially receiving direct gene flow via the Northern current. However, and as in previous studies^8,35^, all haplotypes could be classified in just two haplogroups, ATL and MED. The haplotype coalescence time was estimated at between 113–138 kya and 34–46 kya^8^. This last estimate would correspond to a period before the Last Glacial Maximum, when both haplogroups likely diverged. This period, which was characterized by a sea level decrease of around 120 m^41^, was coupled to a drastic descent of sea temperatures in the North Atlantic, which also produced an intensive cooling of the Alboran Sea^42^. Therefore, both haplogroups may be the result of adaptation to these different environmental conditions, which still exist presently, due to the marked differences in temperature and salinity between Atlantic and Mediterranean waters. Currently, the gene flow consists mainly in the gene content of individuals from the ATL haplogroup entering the Mediterranean across GS.

However, some Mediterranean haplotypes are also reported in Atlantic waters of the Gulf of Cadiz (Table 2). This may be likely due to a much weaker coastal epipelagic counter-current sometimes reported to exit the Mediterranean Sea through the Strait of Gibraltar^13,43^, or to the possible occurrence of advanced zoeal stages in the deeper Mediterranean outflux, which both imply moving a fraction of Mediterranean larvae into the Atlantic. The proportion of ATL frequencies in the Alboran Sea region changed over time (Table2), likely due to seasonal and interannual fluctuations of the current patterns across GS^13,44^. In the present research, GS acted as a strong oceanographic barrier concerning connectivity of *L. depurator* populations (Table S3). This effect was previously detected^8^, but it was not so clear in a later research^35^. For some crustacean, mollusc, and fish species, significant effects of GS on genetic differentiation of populations located at each of its sides have also been reported^45–49^. Not many studies have however yet dealt with on genetic geographical patterns and dispersion in Mediterranean crabs, but among them, Fratini et al.^50^ showed that GS would not act as a barrier reducing gene flow in the intertidal rocky shore marbled crab, *Pachygrapsus marmoratus*, but more recent research would suggest a weak but significant effect^51,52^. For the warty crab *Eriphia verrucosa*, also a rocky shore species, in which a vicariance effect generated during the late Pleistocene across GS was erased due to postglacial expansion and admixture, which restored the gene flow between Atlantic and Mediterranean populations after the last glaciation^53^.

In *L. depurator*, a continental shelf species, a significant decreasing gradient with distance of the ATL haplogroup was found for all studied years from the Gulf of Cadiz eastwards. However, it was not continuous, but instead showed steps likely linked to the precise occurrence of oceanographic fronts. Additionally, significant differentiation was observed in most years between the Alboran Sea populations and the rest of studied western Mediterranean populations. The most probable explanation for the occurrence of this pattern would be the presence of the AOF, considered by many authors as the most relevant barrier generating the interruption or reduction of gene flow between populations of different species^54–58^. It is worth remarking that the AOF is an epipelagic structure, affecting the upper layers of the pelagic environment, where high concentrations of decapod crustacean larvae are usually reported^59,60^. By comparing the annual frequencies of the ATL haplogroup in this area, it was possible to understand the differentiation dynamics between populations. Interestingly, the comparison between WALB and ALAC was significant in all years involved in the temporal series, but it was lost after the FDR correction in 2017 and 2018. Likely, the barrier reducing gene flow between them was the AOF. However, more precise information was obtained in those years when specimens from EALB were available (Table S3 and Fig. 3). According to these results, the effect of the AOF in 2016 would be located between WALB and EALB (Fig. 3a), probably because the East Alboran Gyre was not particularly strong, or was not present, as has been reported in several years^61,62^. In 2017 (Fig. 3b), the AOF was located at its expected, usual location, and both anticyclonic gyres in its structure were well developed^12,19,20^. Conversely, this front would be in its typical place in 2018 (Fig. 3c), but not being especially powerful. Finally, a similar pattern was observed in 2019 (Fig. 3d), where a differentiation, although non-significant, was observed between EALB and ALAC. Thus, for *L. depurator* in the study region, gene flow is not stable over the years and is most probably related to the characteristics of AOF, which intensity and location fluctuate with the oceanographic conditions. In a previous connectivity study of *L. depurator* populations^35^, this front was also reported to act as a barrier for gene flow. However, the AOF did not appear to be a major barrier reducing gene flow in the case of the rocky shore crab *Pachygrapsus marmoratus*^50^ or *Eriphia verrucosa*^52^, as reported above.

When the main Atlanto-Mediterranean current leaves the Alboran Sea, it completes a whole turn along the North African coast, Tyrrhenian Sea, Ligurian Sea, South of France, returning to the Iberian Peninsula from the North, thus circulating in a cyclonic way throughout the remaining studied populations (NCAT, DELTA, VALE and ALAC). Between VALE and ALAC the IC usually allows the current to reach the ALAC area^19^. However, depending on the conjunction of several climatic and oceanographic conditions, in several winters this flux has been shown to turn to the East in this area, thus recirculating to the north along the west of the Balearic Islands^23^. This phenomenon thus generates a barrier at the level of IC which would affect epipelagic larvae in winter-spawning species such as *L. depurator*, thus preventing the connectivity between northern and southern populations, since the main current does not continue southwards, but recirculates to the north at the level of the IC. This phenomenon also allows Atlantic water masses to increase their influence northeast to the ALAC sector. Furthermore, this hydrographic phenomenon is enhanced when the second, eastern gyre in the Alboran Sea is weaker^21^, which is exactly what was observed in 2016 (Fig. 3a). This effect was only detected in this year, thus indicating that IC could have a sporadic, but relevant, incidence on the gene flow between VALE and ALAC populations. This could also be the most plausible explanation for the anomalous genetic composition reported in 2007 in the ALAC population^35^.

Concerning the distribution of *L. depurator COI* haplotypes, taking into account all populations and years, the general overview depicted by the multivariate analysis identified three main areas: Gulf of Cadiz, Alboran Sea, and the remaining Mediterranean populations along the Iberian Peninsula (Fig. 4), a result similar to that reported for the cephalopod *Sepia officinalis*^54^, the seabass *Dicentrarchus labrax*^63^, the red mullet *Mullus barbatus*^47^, and the bivalve *Donax trunculus*^49^. The overlap between the first two areas, the Gulf of Cadiz and the Alboran Sea, is minimal, which indicates the crucial role of GS in separating the two population groups. The Alboran Sea would present a mixture of Atlantic and Mediterranean waters, which is interannually variable depending on the intensity of the two anticyclonic gyres of the Alboran Sea. Therefore, these gyres would determine the exact location and strength of the AOF, acting as a barrier by reducing the interpopulation gene flow.

The present study of *L. depurator COI* haplotypes has allowed to monitor the annual fluctuations of this crucial evolutionary factor. Also informative are the results concerning the third region (the remaining Mediterranean populations studied), characterized by their low proportion of the ATL haplogroup (Table 2). The most outstanding observation is that these populations are not distributed according to their geographical location, and, also, their relative location changes interannually (Fig. 4). Thus, individuals belonging to the ATL haplogroup could have originated from larvae settling after completing the circuit of the main current (a too long trip) or, what would be more reasonable, from the anticyclonic gyres that often detach from the main eastwards current along the northern coasts of Africa, and travel north to the Balearic Islands region south of IC^64^. This last situation would be favoured in those years where the Northern current deviates to the east in the IC^65^. For these reasons, ALAC would be closer to the samples from the Alboran Sea. Also, in some years DELT samples showed a high proportion of the ATL haplogroup. This characteristic could be adaptive to the lower salinity conditions found in this area, due to freshwater runoff supplied by the Ebro River, in a way that the lower salinity present in this area would be closer to that present in Atlantic coastal waters. Although persistent large river runoff may act as a physical barrier to connectivity, as shown for the portunoid crab *Callinectes ornatus*^66^ for the Amazon-Orinoco plume, the large seasonality in Mediterranean river runoff is not strong enough to cause significant genetic differentiation. It is however worth noting that a decrease in salinity is considered a stressful factor for crabs^67^, since osmoregulation implies higher energetic expenses^68–72^. This hypothesis on DELT *L. depurator* crabs deserves to be tested in the future. The population with the highest proportion of MED haplotype is VALE, since it is close to the extreme of the main current circuit and is isolated from the sporadic Atlantic water masses that move northwards from north Africa. This area would therefore present environmental conditions favouring the MED haplotypes.

From these results an interesting question arises: if the ATL haplogroup is constantly penetrating into the Mediterranean Sea, why does it not increase in frequency until it reaches fixation? This would be the expected consequence under a migration model. However, in the studied region, two main evolutionary factors would coexist: migration and selection. Although haplotypes from the ATL group are continuously reaching the Mediterranean populations, they are not so well adapted to the present environmental conditions as those from the MED area. The higher temperature and salinity of Mediterranean waters would be some of the most important environmental conditions to which *L. depurator* individuals need to adapt. This differentiation in ATL and MED haplogroups is most probably the consequence of historic and adaptive processes. In this sense, Cimmaruta et al.^73^ found a genetic differentiation correlated with environmental factors (temperature and salinity) in *Merluccius merluccius*. The possibility of selective differences between ATL and MED haplogroups deserves to be further studied in the future.

Furthermore, under the marine global warming scenario^74–78^ an increase in temperature, and therefore, in changes in other environmental factors, should be expected, generating adverse conditions for ATL *L. depurator* crabs in the Mediterranean. Although population genetics models of migration-selection are complex^79–81^, they deserve to be explored and applied to *L. depurato*r in the Atlantic-Mediterranean transition. Moreover, it would be necessary to follow studies of temporal series to better understand the evolutionary dynamics of these populations.

In summary, *L. depurator* in the Atlantic-Mediterranean transition could be used as a model species with high genetic richness that could contribute to understand the evolutionary dynamics of marine species with similar overall patterns of dynamic biogeography. Furthermore, the information obtained can be useful for properly describing Marine Protected Areas in species with high intraspecific genetic diversity, as well as to develop efficient conservation strategies.

## Materials and methods

### Area of study and sampling procedure

The sampled area included the continental shelf and upper/middle slope from depths around 40 m down to 800 m from the Gulf of Cadiz (Eastern Central Atlantic) to Cape Creus (Northwest Mediterranean Sea). *L. depurator* samples (Fig. 1) were obtained during the MEDITS and ARSA fishery research surveys using standardized demersal trawl gears and sampling protocols^82–84^. Samples were taken yearly in spring from 2014 to 2019 along the entire Mediterranean coast of the Iberian Peninsula (MEDITS surveys), and the Gulf of Cadiz in the Eastern Atlantic (ARSA surveys). All samplings were performed on board the research vessel R/V Miguel Oliver. The sampling gear used in the MEDITS surveys was the experimental bottom trawl gear GOC-73^85^, with a vertical opening of 3 m and a codend stretched mesh size of 20 mm. The ARSA surveys used a Baka 40/60 trawl gear as used in Atlantic ICES fisheries research surveys^83^. Once on board, the total catch was separated by species, counted, and weighed before biological sampling by species taking place. Occurrence and abundance data were then standardized by swept area to obtain information on density and biomass as well as additional fisheries research information.

The seven target populations were selected so that they were located at either side of putative oceanographic barriers (Fig. 1), or by distance criteria in a priori oceanographic homogeneous areas, according to previous knowledge^35^. Thus, the Gulf of Cadiz (CADI) and the Western Alboran Sea (WALB) are separated by the Gibraltar Strait. No apparent major discontinuity is however present between WALB and Eastern Alboran Sea (EALB). The Almeria-Oran Front (AOF) is present between EALB and Alacant (ALAC) sector i.e. between Cape Palos and Cape La Nao. No apparent oceanographic barriers are located between ALAC and the Valencia sector (VALE), located between Cape La Nao and the Columbretes Islands. The exception would be the occasional winter “closure” of the Ibiza Channel, when a frontal oceanographic barrier that occasionally restricts the north–south of the Northern Current that runs along the continental shelf break, forcing it to in a north–south way, and then forces it to return northwards along western Mallorca^65^. The Ebro Delta sector (DELT) expands from the Columbretes islands to north of the Ebro River, on a wide continental shelf area, and is widely affected by the outflow of the Ebro River, which implies a concomitant decrease in salinity throughout the whole area. North of the Ebro Delta, the Catalonia sector (NCAT) constitutes a geomorphologically diverse region between the Ebro Delta and the Pyrenees, with a markedly indented continental shelf with steep submarine canyons in the north, along which the Northern Current flows.

Each population was sampled consecutively each year from 2014 to 2019, except for EALB and NCAT, which could only be sampled in the period 2016–2019, although with usually low sample sizes. In 2019 it was also possible to collect a small sample from the Alboran Island (IALB), located in the middle of the Alboran Sea, between the European and northern African coasts.

### Individual classification, DNA extraction, amplification and sequencing

Given that crabs of different age classes can usually be observed in natural populations and trying to avoid using individuals from different cohorts within the same year, all crabs used for genetic analyses were adult crabs, according to the growth characteristics of this species, suggesting that adult Mediterranean populations strongly depend on the previous year spring recruitment^27,28^. Therefore, only those crabs presenting a carapace width of 41 ± 6 mm were selected, since they would correspond to one year old mature individuals^35^. Accordingly, and for each year, the main phase of dispersion (planktonic larvae) that gave rise to our sampled crabs would have taken place during the previous year winter. Therefore, the oceanographic conditions found on the previous year would be those affecting larval dispersal^35^.

One section of the third or fourth pair of legs was immediately extracted on board, dehydrated, and preserved with 96% ethanol in a labelled vial and stored at 4 °C. Once in the laboratory, DNA was extracted using Chelex resin 10% (Fluka)^86^ (2014–2017 samples) or QIAmp® DNA Mini Kit (Qiagen Inc.) following the provider’s instructions (2018 and 2019 collections). A partial fragment of the *COI* (*Cytochrome Oxidase subunit I*) mitochondrial gene was amplified by PCR using the universal primers LCO1490 and HCO2198^87^. The total volume of the reaction was 20 µl which contained: 1 µl of DNA, 12.7 µl of water (ECOLAV), 4 µl of buffer (× 5) (Promega), 1 µl of MgCl_2_ (25 mM) (Promega), 0.5 µl of dNTPs (1 mM), 0.4 µl of *primer Forward* (LCO1490, 10 µM, 5’-GTCAACAAATCATAAAGATATTGG-3’), 0.4 µl of *primer Reverse* (HC02198, 10 µM, 5’-TGATTTTTTGGTCACCCTGAAGTTTA-3’) and 0.2 µl of GoTaq (5U/µl) (Promega). The amplification protocol was: 4 min at 94 °C, 30 cycles of 1 min at 94 °C, 1 min at 48 °C, 1 min at 72 °C and a final extension of 7 min at 72 °C. PCRs were cleaned with ExoSAP, which is a mixture of 1.2 U of Exonucleasa (Epicentre), and 1.2 U of “Thermosensitive Alkaline Phosphatase” (Promega), and were incubated 15 min at 37 °C and 15 min at 80 °C. DNA sequencing were carried out by Serveis Científics i Tècnics de la Universitat de Barcelona (Barcelona, Spain).

### Analysis of sequences and statistics

All sequences were visually inspected, aligned and trimmed using BioEdit v7.2.6.1^88^ to obtain a final fragment of 527 bp. The number of haplotipes (*Nh*), haplotype diversity (*Hd*) and nucleotide diversity (*π*) was calculated for each population and year using DnaSP v5.10.1 (Librado et al. 2009)^89^. In order to follow the same haplotype nomenclature, in the present work we continued the haplotype series described in^8,35^. Haplotype network was constructed using the Median Joining network algorithm^90^ implemented in the NETWORK v5 software (Fluxus Technology) and phylogenetic tree using Neighbour Joining method of MEGA X^91^. With DnaSP v5.10.1 software^89^, Gamma_ST_ genetic distance values were computed, and their significance (P < 0.05) was calculated using *Snn* statistic^92^. In all cases of multiple comparisons, the FDR correction was applied^93^, and significance was reported for P < 0.05. Geographic distances, to explore the possible correlation between genetic and geographic distances, were obtained with Karto V5.2^94^, following the 200 m depth isobathic line. To obtain this correlation for each year, Mantel tests were calculated. Furthermore, to analyse the distribution of all population samples a PCoA (Principal Coordinate Analysis) was computed. For this study, the only NCAT population used was that of 2019, given the scarceness of collected individuals in the rest of the years. Finally, the dependence of the haplogroup frequency on the geographic distance and the year of capture was analysed using regression models (ANCOVA and linear regression). All these computations were carried out using R language functions^95^.

## Supplementary Information

Supplementary Figure S1.

Supplementary Figure S2.

Supplementary Legends.

Supplementary Table S1.

Supplementary Table S2.

Supplementary Table S3.

## Data Availability

The datasets generated and/or analysed during the current study are available in the GenBank repository, accession numbers: OM241982–OM242947.
