# Supplementary figures and images for: Interannual fluctuations in connectivity among crab populations (Liocarcinus depurator) along the Atlantic-Mediterranean transition
Source: Sci Rep. 2022 Jun 13;12:9797. doi: 10.1038/s41598-022-13941-4 (PMC9192654; doi:10.1038/s41598-022-13941-4)

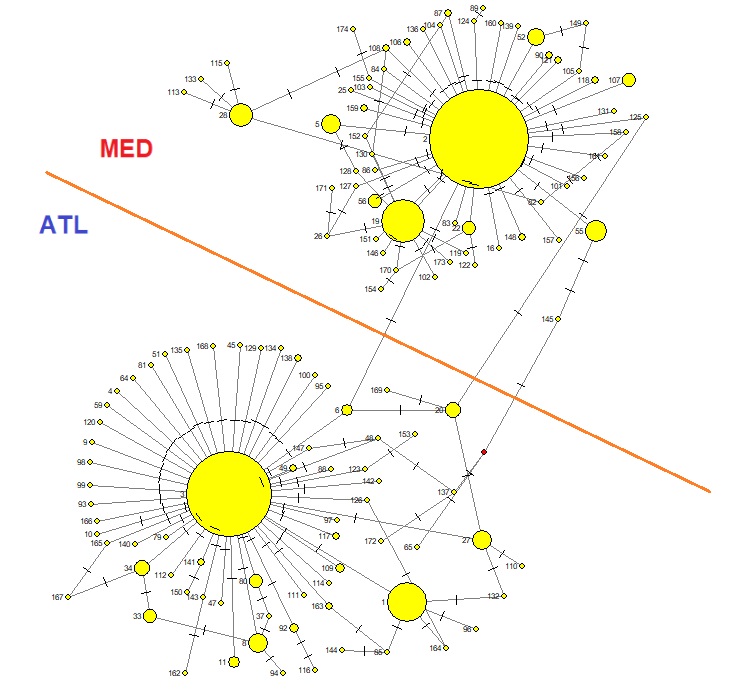

Supplement: Supplementary file 1 — Supplementary Figure S1. [file 41598_2022_13941_MOESM1_ESM.jpg]

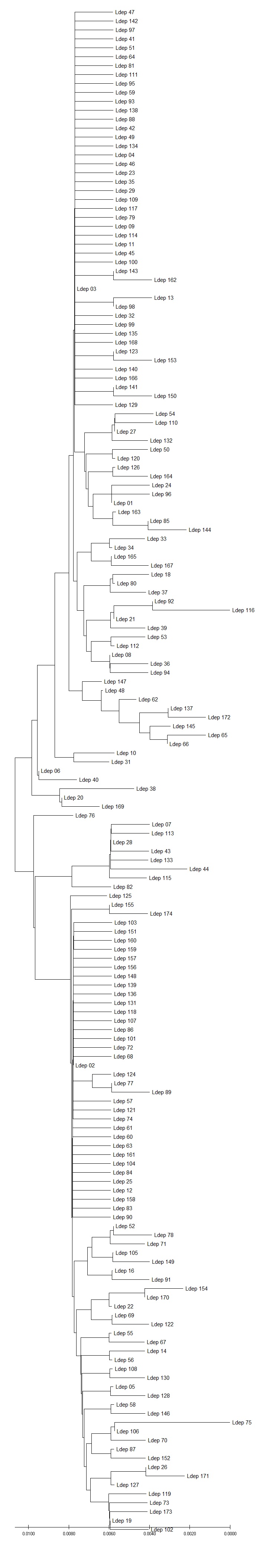

Supplement: Supplementary file 2 — Supplementary Figure S2. [file 41598_2022_13941_MOESM2_ESM.jpg]
