# Supplementary material for: Interannual fluctuations in connectivity among crab populations (Liocarcinus depurator) along the Atlantic-Mediterranean transition
Source: Sci Rep. 2022 Jun 13;12:9797. doi: 10.1038/s41598-022-13941-4 (PMC9192654; doi:10.1038/s41598-022-13941-4)
Supplement: Supplementary file 3 — Supplementary Legends. [file 41598_2022_13941_MOESM3_ESM.docx]

**Supplementary figure legends**

**Figure S1. Haplotype network for the *COI* sequences of *Liocarcinus depurator*.** Each circle represents a haplotype and its size is proportional to its frequency. Transversal small lines in branches indicate one nucleotide change between the connected haplotypes. Haplotype nomenclature: number directly stands for the haplotype (e.g., the haplotype 01 corresponds to Ldep_01). ATL and MED indicate Atlantic and Mediterranean haplogroups, respectively.

**Figure S2. Phylogenetic tree using all different haplotypes detected for the *COI* gene fragment.** It is possible to observe that the first partition separates the haplotypes belonging to ATL and MED haplogroups.
