# Supplementary material for: Interannual fluctuations in connectivity among crab populations (Liocarcinus depurator) along the Atlantic-Mediterranean transition
Source: Sci Rep. 2022 Jun 13;12:9797. doi: 10.1038/s41598-022-13941-4 (PMC9192654; doi:10.1038/s41598-022-13941-4)
Supplement: Supplementary file 4 — Supplementary Table S1. [file 41598_2022_13941_MOESM4_ESM.docx]

| **Population** | **Year** | ***N*** | ***Nh*** | ***Hd*** | ***π (x100)*** |
| --- | --- | --- | --- | --- | --- |
| CADI | 2014  2015  2016  2017  2018  2019 | 27  27  13  19  32  28 | 10  8  9  8  11  11 | 0.761 ± 0.082  0.510 ± 0.116  0.872 ± 0.091  0.766 ± 0.092  0.681 ± 0.091  0.770 ± 0.076 | 0.368 ± 0.074  0.182 ± 0.070  0.535 ± 0.673  0.382 ± 0.063  0.304 ± 0.072  0.310 ± 0.060 |
| WALB | 2014  2015  2016  2017  2018  2019 | 29  30  24  30  30  29 | 14  14  11  12  9  10 | 0.865 ± 0.052  0.857 ± 0.050  0.815 ± 0.063  0.860 ± 0.048  0.791 ± 0.048  0.773 ± 0.057 | 0.380 ± 0.054  0.523 ± 0.064  0.431 ± 0.055  0.524 ± 0.050  0.470 ± 0.042  0.435 ± 0.040 |
| EALB | 2016  2017  2018  2019 | 23  15  31  28 | 8  6  10  9 | 0.581 ± 0.120  0.705 ± 0.114  0.727 ± 0.064  0.717 ± 0.078 | 0.246 ± 0.075  0.296 ± 0.072  0.418 ± 0.053  0.425 ± 0.049 |
| ALAC | 2014  2015  2016  2017  2018  2019 | 30  20  25  28  31  27 | 11  9  7  6  9  8 | 0.740 ± 0.081  0.653 ± 0.122  0.633 ± 0.104  0.487 ± 0.111  0.583 ± 0.102  0.558 ± 0.112 | 0.312 ± 0.064  0.256 ± 0.076  0.301 ± 0.402  0.256 ± 0.067  0.229 ± 0.058  0.215 ± 0.062 |
| VALE | 2014  2015  2016  2017  2018  2019 | 31  29  41  26  30  27 | 7  8  8  5  11  3 | 0.452 ± 0.110  0.554 ± 0.106  0.316 ± 0.095  0.289 ± 0.115  0.605 ± 0.105  0.145 ± 0.090 | 0.144 ± 0.048  0.259 ± 0.070  0.093 ± 0.039  0.128 ± 0.062  0.243 ± 0.071  0.056 ± 0.040 |
| DELT | 2014  2015  2016  2017  2018  2019 | 29  30  25  25  29  24 | 7  6  7  8  9  7 | 0.429 ± 0.114  0.577 ± 0.094  0.617 ± 0.098  0.637 ± 0.105  0.483 ± 0.115  0.558 ± 0.118 | 0.206 ± 0.066  0.253 ± 0.053  0.276 ± 0.084  0.271 ± 0.076  0.144 ± 0.053  0.318 ± 0.089 |
| NCAT | 2016  2017  2018  2019 | 6  1  5  27 | 2  1  1  8 | 0.333 ± 0.215  -  -  0.558 ± 0.112 | 0.063 ± 0.083  -  -  0.204 ± 0.058 |

**Table S1. Sampling population, year of collection, number of analysed individuals (*N*), number of different haplotypes (*Nh*), haplotype diversity (*Hd* ± SD) and nucleotide diversity (*π x 100* ± SD).** The population acronyms are: CADI (Cadiz), WALB (West Alboran Sea), EALB (East Alboran Sea), ALAC (Alacant), VALE (Valencia), DELT (Ebro Delta) and NCAT (North Catalonia). Genetic diversity measures were not computed for NCAT in 2017 and 2018 due to the small sample sizes.
