# Supplementary material for: Interannual fluctuations in connectivity among crab populations (Liocarcinus depurator) along the Atlantic-Mediterranean transition
Source: Sci Rep. 2022 Jun 13;12:9797. doi: 10.1038/s41598-022-13941-4 (PMC9192654; doi:10.1038/s41598-022-13941-4)
Supplement: Supplementary file 5 — Supplementary Table S2. [file 41598_2022_13941_MOESM5_ESM.pdf]

HAPLOTYPE	CADI 14	WALB 14	ALAC 14	VALE 14	DELT 14	CADI 15	WALB 15	ALAC 15	VALE 15	DELT 15	CADI 16	WALB 16	EALB 16	ALAC 16	VALE 16	DELT 16	NCAT 16	CADI 17	WALB 17	EALB 17	ALAC 17	VALE 17	DELT 17	NCAT 17	CADI 18	WALB 18	EALB 18	ALAC 18	VALE 18	DELT 18	NCAT 18	CADI19	WALB 19	EALB 19	IALB 19	ALAC 19	VALE 19	DELT 19	NCAT 19	TOTAL
Ldep_1	1	3	1			2	1		2		1	1	1						3	1		1			3	6		1				4	2	2	1			1		38
Ldep_2	1	4	15	23	22		6	12	20	19	1	6	15	15	34	15	5	3	10	3	20	22	15	1		11	14	20	19	21	5	1	11	14		18	25	16	18	480
Ldep_3	13	10	2	2	2	19	10	1	1	5	5	9	1	2		1		9	5	8	3	1	2		18	7	9	3	1			13	9	6	2	2	1	2	2	186
Ldep_4																										1														1
Ldep_5			1				1	1							1		1						1				1		1	1		1								10
Ldep_6																			1													1								2
Ldep_8	2	1				1								1					2										1											8
Ldep_9																																						1		1
Ldep_10																																1								1
Ldep_11												1							2														1							4
Ldep_16																														1										1
Ldep_19		1	4	2	1		2	1	3	3			2	3	1	5		1	2		1		3		1	1		2	2	1			1	1		2			2	48
Ldep_20		1								1								2														3								7
Ldep_22														2								1					1													4
Ldep_25																												1												1
Ldep_26			1																																					1
Ldep_27	2						1					1						1							1		1					1								8
Ldep_28	3						1				1	1							1		2				2	1	1						1	1						15
Ldep_33														1									1			1														3
Ldep_34	1	1				1	2																		1															6
Ldep_37																													1											1
Ldep_45					1																																			1
Ldep_47												1																												1
Ldep_48		1																																						1
Ldep_49		2																																						2
Ldep_51																				1																				1
Ldep_52			2					1					1										1				1	1		1								1	1	10
Ldep_55			1						1			1	1		1			1															1					2		9
Ldep_56								1															1					1		1						1				5
Ldep_59																									1															1
Ldep_64																																		1						1
Ldep_65																1																								1
Ldep_80							1													1					2															4
Ldep_87																																				1				1
Ldep_90																						1																		1
Ldep_92	2						1																																	3
Ldep_93	1																																							1
Ldep_94	1																																							1
Ldep_95		1																																						1
Ldep_96		1																																						1
Ldep_97		1																																						1
Ldep_98		1																																						1
Ldep_99		1																																						1
Ldep_100			1																																					1
Ldep_101			1																																					1
Ldep_102			1																																					1
Ldep_103				1																																				1
Ldep_104				1																																				1
Ldep_105				1																																				1
Ldep_106				1																																			1	2
Ldep_107					1										1													1	1							1				5
Ldep_108					1																						1													2
Ldep_109					1							1									1																			3
Ldep_110						1																																		1
Ldep_111						1																																		1
Ldep_112						1																																		1
Ldep_113						1																																		1
Ldep_114							1																																	1
Ldep_115							1																																	1
Ldep_116							1																																	1
Ldep_117							1																									1								2
Ldep_118								1																	1															2
Ldep_119								1																																1
Ldep_120								1																																1
Ldep_121									1	1																														2
Ldep_122									1																															1
Ldep_123									1																															1
Ldep_124										1																														1
Ldep_125															1																									1
Ldep_126															1																									1
Ldep_127															1																									1
Ldep_128														1																										1
Ldep_129																1																								1
Ldep_130																1																								1
Ldep_131																1																								1
Ldep_132											1																													1
Ldep_133											1																													1
Ldep_134											1																													1
Ldep_135											1																													1
Ldep_136											1																													1
Ldep_137												1																												1
Ldep_138												1													1															2
Ldep_139													1																											1
Ldep_140													1																											1
Ldep_141																		1												1										2
Ldep_142																		1																						1
Ldep_143																			1																					1
Ldep_144																			1																					1
Ldep_145																			1																					1
Ldep_146																			1																					1
Ldep_147																				1																				1
Ldep_148																					1																		1	2
Ldep_149																							1																	1
Ldep_150																									1															1
Ldep_151																										1														1
Ldep_152																										1														1
Ldep_153																											1													1
Ldep_154																											1													1
Ldep_155																												1												1
Ldep_156																													1											1
Ldep_157																													1											1
Ldep_158																													1											1
Ldep_159																													1								1			2
Ldep_160																														1										1
Ldep_161																														1										1
Ldep_162																																1								1
Ldep_163																																1		1						2
Ldep_164																																	1							1
Ldep_165																																	1							1
Ldep_166																																	1							1
Ldep_167																																		1						1
Ldep_168																																		1						1
Ldep_169																																			1					1
Ldep_170																																				1				1
Ldep_171																																				1				1
Ldep_172																																						1		1
Ldep_173																																							1	1
Ldep_174																																							1	1
TOTAL	27	29	30	31	29	27	30	20	30	30	13	24	23	25	41	25	6	19	30	15	28	26	25	1	32	30	31	31	30	29	5	28	29	28	4	27	27	24	27	966

Table S2. List of haplotipes classified according to population and year. Blue stand for ATL haplogroup, whereas red for MED.
IALB stands for Alboran Is.
