# Supplementary material for: Interannual fluctuations in connectivity among crab populations (Liocarcinus depurator) along the Atlantic-Mediterranean transition
Source: Sci Rep. 2022 Jun 13;12:9797. doi: 10.1038/s41598-022-13941-4 (PMC9192654; doi:10.1038/s41598-022-13941-4)
Supplement: Supplementary file 6 — Supplementary Table S3. [file 41598_2022_13941_MOESM6_ESM.docx]

| **Oceanographic Front** | **Populations compared** | **GammaST** | **Snn** | **P** | **Adjusted P** |
| --- | --- | --- | --- | --- | --- |
| Gibraltar Strait (GS) | CADI_2014 - WALB_2014  CADI_2015 - WALB_2015  CADI_2016 - WALB_2016  CADI_2017 - WALB_2017  CADI_2018 - WALB_2018  CADI_2019 - WALB_2019 | 0.023  0.085  0.020  0.034  0.108  0.100 | 0.556  0.572  0.498  0.619  0.619  0.607 | 0.085  **0.016**  0.781  **0.021**  **<0.001**  **<0.001** | 0.102  **0.032**  0.781  **0.032**  **<0.001**  **<0.001** |
| Almeria-Oran Front (AOF) | WALB_2014 - ALAC_2014  WALB_2015 - ALAC_2015  WALB_2016 - ALAC_2016  WALB_2016 - EALB_2016  EALB_2016 - ALAC_2016  WALB_2017 - ALAC_2017  WALB_2017 - EALB_2017  EALB_2017 - ALAC_2017  WALB_2018 - ALAC_2018  WALB_2018 - EALB_2018  EALB_2018 - ALAC_2018  WALB_2019 - ALAC_2019  WALB_2019 - EALB_2019  EALB_2019 - ALAC_2019 | 0.294  0.181  0.192  0.216  0.010  0.091  0.055  0.294  0.119  0.026  0.054  0.165  0.007  0.118 | 0.699  0.648  0.672  0.622  0.500  0.573  0.628  0.713  0.546  0.531  0.529  0.583  0.433  0.541 | **0**  **0.002**  **0**  **0.004**  0.370  **0.026**  **0.047**  **0**  **0.032**  0.075  0.105  **0.008**  0.960  0.069 | **0**  **0.007**  **0**  **0.011**  0.398  0.052  0.073  **0**  0.056  0.095  0.122  **0.019**  0.960  0.095 |
| Ibiza Channel (IC) | ALAC_2014 - VALE_2014  ALAC_2015 - VALE_2015  ALAC_2016 - VALE_2016  ALAC_2017 - VALE_2017  ALAC_2018 - VALE_2018  ALAC_2019 - VALE_2019 | 0.021  0.012  0.050  0.023  0.008  0.025 | 0.515  0.503  0.603  0.510  0.719  0.526 | 0.162  0.543  **0.006**  0.209  0.717  0.084 | 0.314  0.652  **0.036**  0.314  0.717  0.252 |
| Without discontinuities | VALE_2014 - DELT_2014  VALE_2015 - DELT_2015  VALE_2016 - DELT_2016  VALE_2017 - DELT_2017  VALE_2018 - DELT_2018  VALE_2019 - DELT_2019 | 0.014  0.008  0.043  0.021  0.016  0.059 | 0.473  0.499  0.581  0.531  0.475  0.529 | 0.872  0.381  **0.006**  0.089  0.690  0.119 | 0.872  0.508  **0.048**  0.254  0.789  0.254 |

**Table S3. Effect of oceanographic fronts by studying the genetic distances between populations located in each of their sides.** The population acronyms are the same as in Table S1. Significant P values appear in bold.
